# The Management of Bilateral Ureteric Injury following Radical Hysterectomy

**DOI:** 10.1155/2008/524919

**Published:** 2008-06-18

**Authors:** Matthew B. K. Shaw, Mark Tomes, David A. Rix, Trevor J. Dorkin, Lakkur N. S. Murthy, Robert S. Pickard

**Affiliations:** ^1^Department of Urology, Freeman Hospital, Freeman Road, Newcastle-Upon-Tyne, NE7 7DN, UK; ^2^Department of Uro-Radiology, Freeman Hospital, Freeman Road, Newcastle-Upon-Tyne, NE7 7DN, UK; ^3^Department of Surgical and Reproductive Sciences, University of Newcastle-Upon-Tyne, Newcastle-Upon-Tyne, NE2 4HH, UK

## Abstract

Iatrogenic ureteric injury is a well-recognised complication of radical hysterectomy. Bilateral ureteric injuries are rare, but do pose a considerable reconstructive challenge. We searched a prospectively acquired departmental database of ureteric injuries to identify patients with bilateral ureteric injury following radical hysterectomy. Five patients suffered bilateral ureteric injury over a 6-year period. Initial placement of ureteric stents was attempted in all patients. Stents were placed retrogradely into 6 ureters and antegradely into 2 ureters. In 1 patient ureteric stents could not be placed and they underwent primary ureteric reimplantation. In the 4 patients in which stents were placed, 2 were managed with stents alone, 1 required ureteric reimplantation for a persistent ureterovaginal fistula, and 1 developed a recurrent stricture. No patient managed by ureteric stenting suffered deterioration in serum creatinine. We feel that ureteric stenting, when possible, offers a safe primary management of bilateral ureteric injury at radical hysterectomy.

## 1. INTRODUCTION

Iatrogenic ureteric injury is a well-recognised complication of radical hysterectomy occurring in 5–30% of cases [1, 2]. Bilateral injuries are rare, being documented as isolated case reports but do present a considerable reconstructive challenge [3, 4].

The management of ureteric injury presenting during and following radical gynaecological surgery has been frequently discussed in the literature although the evidence base for such management is restricted to expert opinion, with reports of long-term outcome lacking [5–8]. Injuries recognised during the initial surgery are generally straightforward to treat involving immediate open repair over a ureteric stent. The management of injuries presenting in the postoperative period generally with ureterovaginal fistula formation has evolved over the past decade changing from a predominantly open approach to endourological retrograde or antegrade stent placement [7, 9]. In addition to the ureteric injury it must not be forgotten that pelvic surgery such as radical hysterectomy can affect lower urinary tract function, typically by injury to the pelvic nerves, resulting in a proportion of women experiencing long-term bladder dysfunction [10].

Issues surrounding the management of bilateral ureteric injury are more complex and are less considered in the literature despite the challenging reconstructive problem that they present. The standard methods of surgical management used for unilateral injury may need to be modified or used in combination for cases of bilateral injury and close observation is needed to minimise further loss of renal function and to avoid uro-sepsis.

In view of the rarity of bilateral ureteric injury and the lack of literature outlining the management of such cases, we conducted a chart review of 5 women who attending our tertiary referral urology department for treatment to illustrate the salient points of diagnosis and management of these complex injuries.

## 2. MATERIALS AND METHODS

A retrospective analysis of a prospectively acquired departmental database of ureteric injuries was performed for the years 1999 to 2005. Five patients with bilateral ureteric injury occurring during radical hysterectomy were identified. The case notes and imaging of the five patients were comprehensively reviewed.

All patients underwent imaging immediately following referral in the form of intravenous urography (IVU) followed by retrograde ureteropyelography. Surgical operation notes and inpatient stays were all reviewed. Follow up information reviewed included outpatient consultation, IVU, isotope renography, retrograde studies, and cystometry.

Patients were included in the study if the injury to the ureters was bilateral and occurred at the time of radical hysterectomy for malignant cervical pathology. Patients were excluded if they had undergone preoperative chemotherapy or radiotherapy or if the injury only became apparent after the subsequent use of these treatment modalities; this was in order to standardise the aetiology of the ureteric injury.

## 3. RESULTS

### 3.1. Presentation

Five patients referred with bilateral ureteric injury following radical hysterectomy for cervical cancer were identified from the database. The median age (range) at the time of hysterectomy was 42 (38–84) years. In four cases the main presenting symptom was that of a vaginal urinary leak whilst the fifth patient presented with anuria associated with acute renal failure on biochemical assessment. The median time (range) from hysterectomy to discovery of the ureteric injury was 21 (12–58) days. Follow-up was available for a median (range) of 26 (21–88) months. The findings and their progress are summarised in Table 1.

The diagnosis of ureteric injury was made by intravenous urography and biochemical analysis of the vaginal effluent in the four patients presenting with a vaginal urine leak. The anuric patient was initially investigated by noncontrast CT urography after an ultrasound examination had suggested upper urinary tract dilatation. CT confirmed bilateral hydroureteronephrosis down to the pelvic ureter with a large pelvic fluid collection believed to be a urinoma.

**Figure 1:**
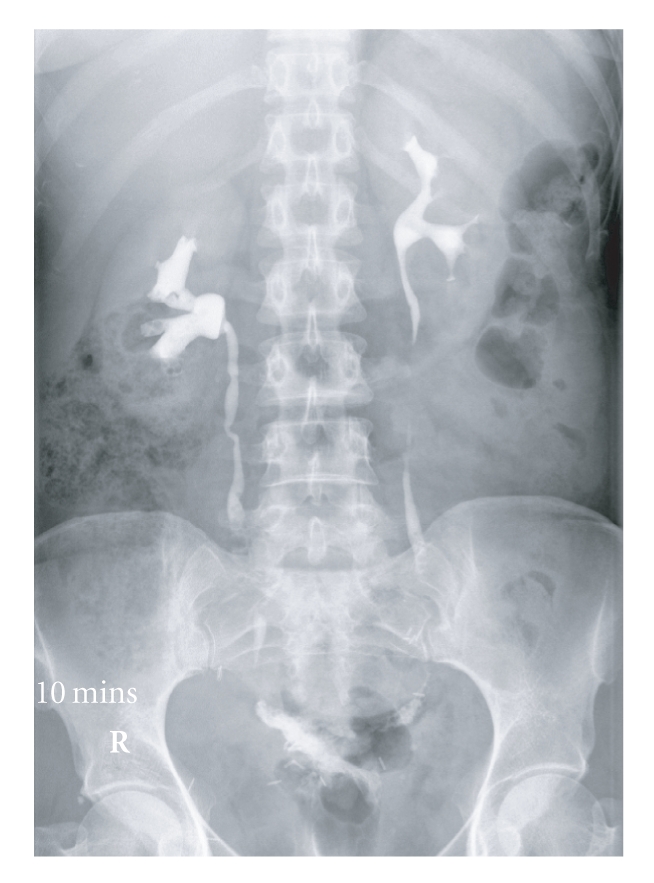
Intravenous urogram showing bilateral ureterovaginal fistula and significant quantities of gas within both ureters.

After initial radiological imaging suggesting ureteric injury (see Figure 1), all went on to have cystoscopy and bilateral retrograde ureteropyelography performed. All 10 ureters demonstrated stenotic defects in the distal pelvic segment on initial contrast injection. Further contrast injection supplemented by methylene blue leak test showed that 4 women had ureterovaginal fistulae arising from 6 of the 8 ureters with the remaining 2 ureters have stenoses without leakage. In the final patient, both ureters were draining into a pelvic urinoma. This same patient was also found to have an associated vesicovaginal fistula at the time of repeat cystoscopy.

### 3.2. Management

The initial management option attempted in all 5 cases was retrograde placement of a ureteric stent. If this was unsuccessful, the procedure was repeated using an antegrade approach via a percutaneous nephrostomy. Using these methods, four patients were initially managed by ureteric stenting alone; 6 ureters using a retrograde approach and 2 ureters using an antegrade approach. The remaining patient could not be stented either in an antegrade or retrograde fashion due to an inability to negotiate the strictured portion of the ureter and therefore primary open repair by bilateral ureteroneocystotomy with a unilateral psoas hitch was performed.

### 3.3. Follow-up

Of the 4 patients initially managed with ureteric stents, 3 patients demonstrated complete bilateral ureteric healing, with no stricture formation on retrograde ureteropyelography and therefore had stents removed at 8, 16, and 24 weeks, respectively. In the remaining stented patient, the ureterovaginal fistula persisted and open ureteroneocystotomy with closure of the fistula was performed 22 weeks after initial stent insertion.

Of the 3 women initially managed by endourological methods alone, one has, since developed a stricture at the site of the original injury requiring balloon dilatation and ureteric stent insertion. This stricture, however, persisted and a subsequent ureteroneocystotomy was performed. Imaging by means of IVU and renography at a median 18 months shows only one of the 6 ureteric units to be minimally dilated in these women. This patient has decided not to pursue further invasive management.

The technique used for the two patients who required early open reconstruction was to pass the freed left ureter under the sigmoid mesocolon and then perform separate reimplantation of both ureters into a right psoas bladder hitch. The ureters were implanted through a short detrusor tunnel and bladder mucosa groove without special precaution to prevent reflux. On follow-up, the patient showed neither evidence of reflux nor of obstruction.

Four of the 5 patients currently have normal serum creatinine. The remaining patient, who underwent immediate open reconstruction, presently has a stable elevation of her creatinine at 177 *μ*mol/L; this has not changed significantly over 48 months of follow-up.

Cystometry (CMG) was performed on four of the five patients. In two of the patients the indication was symptomatic urge incontinence when no such symptoms existed prior to the ureteric injury and detrusor overactivity was present in both. In the two other patients symptomatic voiding difficulties prompted the CMG studies; one study suggested low pressure voiding, with voiding to empty and the other study was normal.

## 4. DISCUSSION

This series of 5 cases demonstrates that primary management by stenting can safely be accomplished for most (80%) women with bilateral ureteric injury following radical hysterectomy. This approach has the advantage of stabilising the situation, protecting renal function, and drying up the vaginal leakage, whilst the patient recovers from the primary surgery. The potential disadvantage exists however of needlessly delaying definitive open repair. Stenting was all that was needed for 2 of the cases with 2 women requiring delayed open repair. Despite this endourological success, vigilance is required to detect silent ureteric stenosis in the longer term, which may threaten remaining renal function [11]. Open surgery can therefore be reserved for the case where stenting fails or for later management of persistent distal ureteric strictures. Open repair in these cases is hampered by the difficulty in performing bilateral bladder reconfiguration by psoas hitch or Boari flap. We overcame this hurdle by swinging the mobilised left ureter to the right iliac fossa allowing separate implantation of both injured ureters into the same bladder flap.

Current surgical management of cervical carcinoma mandates wide excision of the local disease and completes removal of the draining lymphatics [12]. Despite the ureters being identified and protected during the dissection, it is easy to severely compromise the blood supply, leaving a devascularised segment which then stenoses and ruptures in the immediate postoperative period. This mechanism of injury explains the delayed presentation and the universal finding of a stenosed distal segment in our series. This is in line with published results which suggest that between 5% and 30% of women undergoing radical hysterectomy suffer a ureteric injury [1] and of these, 85% involve the distal ureter [5]. Despite improving surgical technique and increased awareness of the risks of ureteric injury, it is likely to be a problem that will continue to challenge the urological surgeon. Management of these patients is often taxing requiring a range of technical skills and is hampered by the lack of clear evidence supporting one particular treatment modality and the medicolegal pressures that surround patients with iatrogenic injury.

Conventional management of ureteric injury presenting in the postoperative period has been by open surgery, particularly when faced with ureterovaginal fistula [13]. With more recent developments in endourological techniques and equipment, the use of ureteric stenting as a primary manoeuvre is amassing an increasing volume of supportive evidence. Support for the use of ureteric stents is provided by Selzman and Spirnak, who reviewed the management of ureterovaginal fistulas treated at their institution over 20-year period [7]. Seven patients whose stents were successfully placed and left in situ for a long enough time period to allow fistula closure all showed complete healing. One patient developed a stricture that required further endourological intervention. Giberti et al. also produced excellent early results from the successful stenting of ureteric injuries, however, three of their cases went on to have open reconstruction [5]. The use of ureteroscopy in assisting stent placement has been shown to be successful even in the face of previously failed attempts [14]. In a series of patients with 10 injured ureters, Tsai et al. report the combined use of a ureteroscope and a fluoroscopically guided antegrade snare to place a ureteric stent [15]. In this study, six required no further intervention, and three required balloon dilatation for subsequent stricture formation. In one case (10%) balloon dilation was unsuccessful and open ureteric reimplantation was required.

An obvious area of concern in bilateral ureteric injury is the preservation of renal function over both the short- and long-terms. In respect of this, the evidence comparing open reconstruction and endourological methods is scant. Indeed some studies deem bilateral injury an indication to exclude these patients from endourological treatment and proceed directly to open reconstruction [6], however in our study no patient had a rise in plasma creatinine from the time subsequent to stent placement.

In our study, we demonstrate that initial endourological management offers a safe, minimally invasive option in patients with bilateral ureteric injury. The placement of ureteric stents allows recovery from a large and traumatic procedure. In many cases, ureteric stenting offers a definitive treatment and avoids further extensive and challenging procedures.

## Figures and Tables

**Table 1:** Details of diagnosis, management, and outcome of patients with bilateral ureteric injury. Abbreviations: IVU (intravenous urogram), CT scan (computed tomogram).

Patient	Presenting symptom	Initial imaging	Ureterovaginal fistula	Antegrade/retrograde stent	Healed with stent alone	Long-term outcome
1	Vaginal discharge	IVU	Yes, 1 ureter	Retrograde, both ureters	Recurrent right stricture, reimplanted	Normal creatinine, detrusor overactivity
2	Vaginal discharge	IVU	Yes, 2 ureters	Retrograde + antegrade	Yes	Normal creatinine, endoscopic dilatation stricture
3	Anuria & acute renal failure	Ultrasound & CT scan	No	Unable to stent, therefore reimplantation	—	Elevated creatinine, detrusor overactivity
4	Vaginal discharge	IVU	Yes, 2 ureters	Retrograde + antegrade	Persistent leak, therefore reimplantation	Normal creatinine, low-pressure voiding
5	Vaginal discharge	IVU	Yes, 1 ureter	Retrograde, both ureters	Yes	Normal creatinine, hydroureter (1)

## References

[1] Ralph G, Tamussino K, Lichtenegger W (1988). 19 Urological complications after radical abdominal hysterectomy for cervical cancer. *Baillière's Clinical Obstetrics and Gynaecology*.

[2] Ku JH, Kim ME, Jeon YS, Lee NK, Park YH (2003). Minimally invasive management of ureteral injuries recognised late after obstetric and gynaecologic surgery. *Injury*.

[3] Rafique M, Arif MH (2002). Management of iatrogenic ureteric injuries associated with gynecological surgery. *International Urology and Nephrology*.

[4] Liapis A, Bakas P, Giannopoulos V, Creatsas G (2001). Ureteral injuries during gynaecological surgery. *International Urogynecology Journal*.

[5] Giberti C, Germinale F, Lillo M, Bottino P, Simonato A, Carmignam C (1996). Obstetric and gynaecological ureteric injuries: treatment and results. *BJU International*.

[6] Menez R, McGinty DM (1978). The management of delayed recognized ureteral injuries. *The Journal of Urology*.

[7] Selzman AA, Spirnak JP (1996). Iatrogenic ureteral injuries: a 20-year experience in treating 165 injuries. *The Journal of Urology*.

[8] Carley ME, McIntire D, Carley JM, Schaffer J (2002). Incidence, risk factors and morbidity of unintended bladder or ureter injury during hysterectomy. *International Urogynecology Journal*.

[9] Gül O, Eroölu M, Beyribey S (2001). Repair of bilateral complete ureteral ligation that occurred during hysterectomy. *International Urology and Nephrology*.

[10] Ito E, Saito T (2004). Nerve-preserving techniques for radical hysterectomy. *European Journal of Surgical Oncology*.

[11] Shapiro SR, Lebowitz R, Colodny AH (1975). Fate of 90 children with ileal conduit urinary diversion a decade later: analysis of complications, pyelography, renal function and bacteriology. *The Journal of Urology*.

[12] Pieterse QD, Maas CP, ter Kuile MM (2006). An observational longitudinal study to evaluate miction, defecation, and sexual function after radical hysterectomy with pelvic lymphadenectomy for early-stage cervical cancer. *International Journal of Gynecological Cancer*.

[13] Hoch WH, Kursh ED, Persky L (1975). Early, aggressive management of intraoperative ureteral injuries. *The Journal of Urology*.

[14] Reddy CJ, Heman-AchKah CA, Gelister JSK (1997). Ureteroscopic salvage of a uretero-vaginal fistula. *BJU International*.

[15] Tsai CK, Taylor FC, Beaghler MA (2000). Endoscopic ureteroureterostomy: long-term followup using a new technique. *The Journal of Urology*.

